# Distribution of G6PD deficiency genotypes among Southeast Asian populations

**DOI:** 10.1186/s41182-021-00387-z

**Published:** 2021-12-20

**Authors:** Indah S. Tantular, Fumihiko Kawamoto

**Affiliations:** 1 Institute of Tropical Disease, Universitas Airlangga, Surabaya, Indonesia; 2 Department of Parasitology, Faculty of Medicine, Universitas Airlangga, Surabaya, Indonesia; 3 Department of Environmental and Preventive Medicine, Oita University Faculty of Medicine, Yufu, Japan

**Keywords:** Glucose-6-phosphate dehydrogenase, G6PD deficiency, Genotype, Distribution, Southeast Asian population

## Abstract

Glucose-6-phosphate dehydrogenase (G6PD) deficiency is a group of X-linked, hereditary genetic disorders caused by mutations in the G6PD gene and results in functional variants of about 400 biochemical and clinical phenotypes. Among them, more than 215 genotypes have been identified so far. In this review, specific features of the genotype distribution in different communities and countries are discussed based on multiple reports and our molecular epidemiological studies of Southeast Asian countries. Particularly, in Indonesia, the frequency distribution of G6PD deficiency variants was distinct between western and eastern Indonesian populations, suggesting two different gene flows during Indonesian expansions.

## Background

Glucose-6-phosphate dehydrogenase (G6PD) deficiency is a group of X-linked, hereditary genetic disorders caused by mutations in the G6PD gene. These mutations result in protein variants with decreased levels of enzyme activities and are correlated with a wide range of biochemical and clinical phenotypes [1]. The G6PD gene spans 18 kb on the X chromosome (Xq28), contains an open reading frame of 1545 base pairs and encodes 13 exons and 12 introns. Most instances of G6PD deficiency are caused by a single nucleotide mutation resulting in one amino acid change among 515 amino acids. To date, more than 400 biochemical variants have been described, and more than 215 mutations among them have been discovered at the molecular level [2–4].

In Southeast Asia, there is significant heterogeneity of the dominant variants in different areas, which appears to be closely related to the geographic locations and associated with different racial and ethnic groups [5, 6]. Since 1997, we have been surveying malaria and G6PD deficiency in malaria endemic areas in Southeast Asian countries [6–13] using two rapid diagnostic methods [14, 15]. With these methods, malaria patients can be informed of the diagnostic results on-site, usually within 30 min of a blood examination, and they are prescribed antimalarial drugs, including primaquine if their G6PD activity is normal. Blood samples collected from G6PD-deficient volunteers were analyzed at the molecular level, and distributing variants of G6PD deficiency were identified in each population. All G6PD-deficient blood samples obtained in our field surveys were confirmed as having deficient genotypes without exception. In this review, we show the precise distribution and distinct features of the distribution of G6PD deficiency genotypes among several populations in Southeast Asia by referring to published reports.

### Distribution of G6PD deficiency genotypes in Vietnam, Laos and Cambodia

Four studies on Vietnam [6, 12, 16, 17] reported G6PD Viangchan (871G > A, 1311C > T, IVS11 nt93T > C; WHO Class II) as the most dominant variant in the Kinh population (largest ethnic group; 164/275, 59.6%) and in two minority populations, K’Ho and Stieng (19/41, 46.3%) (Table 1). This variant is also very common in the Lao (245/298, 82.2%) and Khmer (637/675, 94.4%) populations of Laos and Cambodia, respectively. In the Kinh population, variants of Chinese origins, such as G6PD Kaiping (1388G > A; WHO Class II), G6PD Canton (1376G > T; WHO Class II), G6PD Chinese-5 (1024C > T; WHO Class III), G6PD Quing Yuan (or Chinese-4; 392G > T; WHO Class III), and G6PD Gaohe (95A > G; WHO Class III), were also detected (45/275, 16.4%). In addition, G6PD Union (1360C > T; WHO Class II), which originated from Oceania, was also observed, suggesting that the Kinh population has many different ancestral sources from continental Southeast Asia, China and Oceania. Indeed, it is well known that Vietnam had a strong historical connection with China for more than 2000 years. Until recently, marriages between the Kinh and Chinese were common, which would allow G6PD variants of Chinese origins, such as G6PD Viangchan, being introduced into the Kinh population, reflecting another ancestral source for Southeast Asian populations. However, these Chinese variants are very rare or absent in the K’Ho and the Stieng populations, suggesting that these minorities remained in remote areas, as seen in the present day, and/or that they rejected relations or marriages with other tribes or foreigners. This tendency is also seen in Lao and Khmer ethnic groups, which would indicate a more simple and homogeneous ancestry than the Kinh ethnic group.

**Table 1:** Frequency distributions of G6PD variants reported from Southeast Asian countries

Country	Vietnam		Laos	Cambodia		Thailand	Myanmar		Malaysia		Indonesia		Philippines
Ethnicity	Kinh	Others	Lao	Khmer	Others	Thai	Burman	Others	Malay	O.Asli	Java	Eastern	
Viangchan (871G > A)	164	19	245	637	5	169	9	3	36	6	7	31	6
Mahidol (487G > A)			7	10		97	701	260	18		2		
Vanua Lava (383 T > C)									3			95	1
Mediterranean (563C > T)				5		1	2		25		5	2	
Coimbra (592C > T)	2			6			3		4	5	1	11	
Chatham (1003G > A)									2			31	8
Kaiping (1388G > A)	14		7			50	13	10	2			30	
Canton (1376G > T)	13		8	9		38	16	2	4		4		
Chinese-5 (1024C > T)	11	1		3		3						2	
Quing Yuan (392G > T)	5		6			7	8	8					
Chinese-3 (493A > G)													4
Gaohe (95A > G)	2					3							
Union (1360C > T)	9		25	4		19	4		2				38
Others	5			1		1	6		2				
1311T/93C*	50	21				9		95	3	57	5		1
Total	275	41	298	675	5	397	762	378	101	68	24	202	58

Data of Vietnam are reported from [6, 12, 16, 17], and Others include K’Ho and Stieng minorities. Data of Laos are reported from [7, 17–20] and from Laotian immigrants in Hawaii [21]. Data of Cambodia are reported from [10, 17, 19, 22–24], and Others include 3 minorities. Data of Thailand are reported from [17, 25–31]. Data of Myanmar are reported from [5, 7, 9, 17, 19, 28, 32–36] and Others included 6 minorities. Data of Malaysia are reported from [7, 37–40]. Data of western Indonesia are reported in Javanese, Betawi and Sundanese [7, 41, 42]. Data of eastern Indonesia are reported from [7, 8, 11, 13, 43]. Data of the Philippines are reported from [21, 44, 45]. * G6PD 1311T/93C

G6PD 1311C > T with IVS11 nt93T > C (designated here as G6PD 1311T/93C; WHO Class III) was found as another common genotype in Vietnam (71/316, 22.5%). This mutation was also detected among the Kinh and the Stieng populations. G6PD 1311T/93C is a silent mutation that does not change any amino acids in exon 11, but it causes lower G6PD activity by an unknown mechanism. It was reported first in Filipinos [44], and later, many cases were discovered in populations of the Javanese [41], Chinese [46], southern Thai [26], Malaysian Malay [38], Orang Asli (Malaysian aborigine [38–40]) and Kachin (a minority in Myanmar [5]). The Orang Asli is one of the “Proto”-Malay groups and well known as an ancient ethnic group in Southeast Asia, suggesting this variant has existed since antiquity along with G6PD Viangchan. Quite interestingly, however, G6PD 1311T/93C is not distributed in Laos and Cambodia despite their geographical continuity.

G6PD Viangchan is always accompanied with mutations at 1311C > T in exon 11 and IVS 11 nt93T > C in intron 11, whereas G6PD Jammu (871G > A; WHO Class II), which is dominantly distributed in India, has the wild type sequence at 1311C with IVS11 nt93T. These two variants are thought to have arisen independently [47]. It is worth noting that G6PD Mediterranean (563C > T; WHO Class II) also has two subtypes at polymorphisms 1311 and IVS 11 nt93: The Mediterranean subtype accompanying “1311C > T with IVS11 nt93T > C” and the Indo-Pakistan subtype with the wild type sequence “1311C with IVS11 nt93T (see below).

Among the Lao population in Laos, the frequency distribution of G6PD Union is fairly high (25/298, 8.4%) in comparison with the Kinh (9/275, 3.3%) or the Khmer (4/675, 0.6%) populations for unknown reasons. G6PD Union is dominantly detected from countries in the Pacific Ocean such as the Philippines [21, 44], Papua New Guinea [48], Solomon Islands [49] and Vanuatu archipelago [50]. It is probable that a high prevalence of this variant in the Lao population may be related to foreign migrant workers from the Philippines.

Recently, an analysis of G6PD genotypes in the Lao Theung (or the Lao Mon-Khmer) population was reported from Laos [51]. This tribe is the second largest ethnic group, and their language (the Mon-Khmer language) is different from the Lao ethnic group, who speaks languages belonging to the Thai-Kra Dai family. Surprisingly, the most dominant variant in the Lao Theung tribe is G6PD Aures (143T > C; WHO Class III; 17/29, 58.6%), which is commonly detected in Mediterranean and North African populations. Next common are G6PD Union (3/29, 10.3%) and G6PD Jammu (2/29, 6.9%), but G6PD Vianghan was rare (1/29, 3.4%) among this tribe. All these results taken together strongly suggest that the Lao Theung tribe has different ancestral origins from the Lao ethnic group [51].

### G6PD deficiency genotypes in Thailand, Myanmar, Malaysia and the Philippines

Among the Thai population, G6PD Viangchan is also the most common variant (169/397, 42.6%), followed by various genotypes of Chinese origins (101/397, 25.4%), G6PD Mahidol (487G > A; WHO Class III; 97/397, 24.4%) and G6PD Union (19/397, 4.8%). Therefore, the frequency distribution of G6PD variants in the Thai population is quite similar to that in the Kinh population, except for the high prevalence of G6PD Mahidol. As in the Kinh group, the Thai population is derived from complicated ancestries that involve intermarriages with other tribes or foreigners.

In Myanmar, G6PD Viangchan is very rare (12/1140, 1.1%). G6PD Mahidol is the most dominant in the Burman population (701/762, 92.0%) and in many minority populations, such as the Mon, Karen, Rakhine, Shan, etc. [5, 7, 17, 31–33]. Because these minorities have their own languages, the presence of new genotypes or distinct distribution patterns of variants was anticipated, as seen in the Lao Theung population in Laos. However, almost variants detected from these minorities were G6PD Mahidol only [7]. This observation indicates that, in spite of their different cultural backgrounds, these populations probably share the same ancestry origin with the Burman group before migration from southern China to the present territories. It is interesting to note that this variant was entirely absent in Vietnam and rare in Laos (7/298, 2.3%) and Cambodia (10/675, 1.5%). This observation suggests that all these ethnic groups have different origins from each other. In Myanmar, a high prevalence of G6PD 1311T/93C (95/182; 52.2%) was reported among the Kachin ethnic group and only this group for reasons unknown [5].

In Malaysia, Malaysian Malay, Chinese and Indian people make up the three major ethnic groups. In addition to these groups, minor indigenous communities called Orang Asli inhabit the Malay Peninsula. In the Malay population, G6PD Viangchan (36/101, 35.6%), G6PD Mahidol (18/101, 17.8%) and G6PD Mediterranean (25/101, 24.8%) are most common, followed by G6PD Coimbra (592C > T; WHO Class II) and G6PD Chatham (1003G > A;WHO Class II). A few cases of G6PD Vanua Lava (383 T > C; WHO Class II; a variant reported first from Melanesia [50]) and G6PD Union have also been identified. Regarding G6PD Mediterranean, the Malay population possess both subtypes [38]: The Mediterranean subtype (563 C > T with 1311T/93C), which may have originated from Mediterranean countries, and the Indo-Pakistan subtype (563 C > T with the wild sequence of 1311C/93T), which may have originated from India independently.

G6PD Coimbra is one of the oldest variants originated from Europe and is found in several Asian countries, including Vietnam, Cambodia, Myanmar, Malaysia, and the Orang Asli population. In contrast to the wide distribution of G6PD Coimbra, however, another old variant of Europe origin, G6PD Chatham, is found only in the Malaysian Malay among Southeast Asia populations. This variant as well as the two subtypes of G6PD Mediterranean may have been introduced to Malaysia by European people and/or Indian immigrants who settled through British colonization. On the other hand, variants of Chinese origins in the Malay population are rare (6/101, 5.9%) when compared with the Kinh (45/275, 16.4%) and Thai (101/397, 25.4%) populations. This observation may reflect Malay and Chinese people not intermarrying, possibly for religious reasons.

The frequency distribution of G6PD genotypes in the Philippines is remarkably different from continental Southeast Asian populations and Indonesian populations in that it has the G6PD Union as the most common variant (38/58, 65.5%). One case of G6PD Vanua Lava was reported from a Filipino immigrant boy in Spain [45].

### G6PD deficiency genotypes in Indonesia

**Fig. 1:**
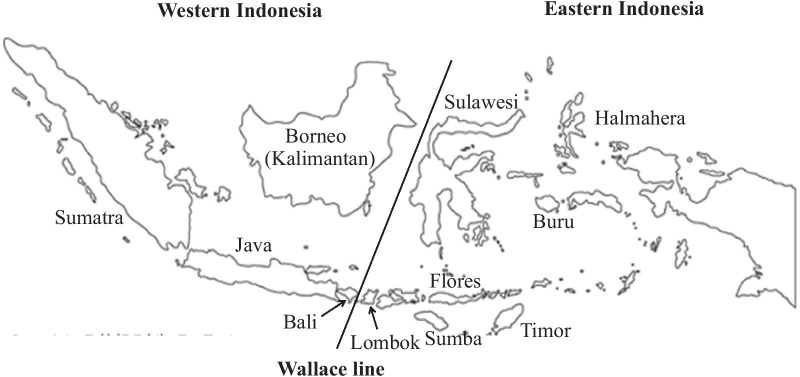
The Indonesian archipelago separated into western and eastern parts by the Wallace line

According to wildlife belonging to Asia and to Australia, the Indonesian archipelago is divided mainly into two parts, western and eastern Indonesia, by the Wallace line [52] at the Lombok Strait between the Bali and Lombok islands (Fig. 1). During the last Ice Age, all the islands of western Indonesia up to Bali were linked to Southeast Asian continent and created a unique peninsula named “Sundaland”, where the “Deutro”-Malay people were able to walk between the Asian continent and the islands of Sumatra, Java, Borneo (Kalimantan) and Bali. Therefore, genetic interactions may have arisen among the Deutro-Malay population, and similar genes flows, including G6PD genes, may have been shared among the Malaysian Malay and western Indonesian populations such as the Sumatra Malay, Sundanese, Javanese, Balinese, etc.

With respect to the molecular analyses of G6PD genotypes among western Indonesian populations, 24 cases of Java islanders (17 Javanese, 6 Betawi and 1 Sundanese) have been reported so far (Tables 1 and 2; [7, 41, 42]). The presence of G6PD Viangchan, G6PD Mahidol, G6PD Mediterranean and G6PD 1311T/93C in these Java islanders may imply a resemblance to the frequency distribution of the Malaysian Malay population (Table 2).

**Table 2:** Distribution of G6PD genotypes reported in the Malaysian Malay and western and eastern Indonesian populations

	Western				Eastern Indonesia						Subtotal
Island	Malaysia	Java	Sulawesi	Bangka	Sumba		Flores		Timor	Halmahera/Buru	
Ethnicity	Malay	3 tribes	4 tribes	Sangirese	Sumba	Savu	6 tribes	Sikka	Timor	Ambonese	
Viangchan (871G > A)	36	7		1	24	1	4	1			31
Mahidol (487G > A)	18	2									
Vanua Lava (383T > C)	3		5	3	53	3	13	3	4	11	95
Mediterranean (563C > T)	25	5					2				2
Coimbra (592C > T)	4	1					2	9			11
Chatham (1003G > A)	2				14		1	16			31
Kaiping (1388G > A)	2				1	1	5	23			30
Canton (1376G > T)	4	4									
Chinese-5 (1024C > T)							2				2
Union (1360C > T)	2										
1311T/93C	3	5									
Total	99	24	5	4	92	5	29	52	4	11	202

Data of Java islanders are reported from [7, 41, 42] and data of eastern Indonesian populations from [7, 8, 11, 13, 43]

In eastern Indonesia population, however, the most common variant is G6PD Vanua Lava (95/202, 47.0%), which is unlike the Java islanders (Table 2). This genotype was first reported in Vanuatu [50] and found later to spread from Papua New Guinea [48] to eastern Indonesia [7], Malaysia [38] and the Philippines [45] (Table 1). This variant was detected as the most dominant genotype in all eastern Indonesian islands, including the Halmahera, Buru, Flores, Sumba, West Timor, Banka and Sulawesi islands (Table 2), and it accounts for nearly half of all detected variants. However, G6PD Vanua Lava, as well as G6PD Chatham, was never detected in continental Southeast Asia populations except the Malaysian Malay population (Table 1). On the contrary, G6PD Mahidol, which is widely distributed from Myanmar, Thailand, Malaysia to Java, did not reach the eastern Indonesia islands or the Philippines. Furthermore, G6PD Union and G6PD 1311T/93C were also not detected in eastern Indonesian populations, and G6PD Mediterranean and Chinese genotypes are very rare or absent (except for G6PD Kaiping in the Sikka population in the Flores island). It is somewhat strange that the G6PD Union mutation was never observed in eastern Indonesian populations, despite the possible connection between this population and Filipino and Melanesian groups. A probable reason for the lack of G6PD Union in eastern Indonesian populations is the genetic drift caused by their long-term isolation [7].

All these results strongly suggest that the frequency distribution of G6PD variants in Indonesia may differ greatly between western and eastern populations. The separation of distributed G6PD variants between western and eastern Indonesia was also proposed by a bioinformatics analysis [53] using the data cited in this review. To support this hypothesis further, we conducted field surveys for G6PD deficiency (unpublished) in a western Indonesian tribe, the Balinese group of Bali Island, and an eastern Indonesian tribe, the Sasak group of Lombok Island, and compared the distributions of G6PD variants. The Bali and Lombok islands face across the Wallace line from the east side of the western Indonesian islands and the west side of the eastern Indonesian islands, respectively (Fig. 1).

As expected, G6PD Vanua Lava was found to be the most dominant variant in the Sasak population (*n* = 18; 9 G6PD Vanua Lava + 7 G6PD Viangchan + 2 G6PD Chatham). In contrast, this variant was absent in the Balinese population (*n* = 9; 4 G6PD Mediterranean + 3 G6PD Viangchan + 2 G6PD Chatham). However, the sample size for the Balinese population was too small, meaning more field surveys of this island are needed. Nevertheless, these preliminary results support our hypothesis that G6PD Vanua Lava is distributed in eastern Indonesian populations but not in western populations, implying two different gene flows during the expansions of the Indonesian peoples: In the western Indonesian islands, the Deutro-Malay populations had genetic interactions without involvement of the G6PD Vanua Lava mutation, while eastern Indonesian islanders, who carried this mutated gene, spread around eastern Indonesian islands and Melanesia.

According to a recent new theory, the migration and expansion of Melanesian (Austronesian language speakers) into the Pacific Ocean [54, 55] are thought to have begun with Taiwan aborigines, a type of Austronesian group, who expanded from Taiwan to the Pacific through the Philippines, Sulawesi and other Indonesian islands. Therefore, there is a possibility that Taiwan aborigines are also the origin of eastern Indonesian islanders. G6PD deficiency genotypes in the Taiwan aboriginal tribes were investigated by Tang et al. [56], who identified 5 cases of G6PD Coimbra and 6 cases of G6PD Chinese-3 and G6PD Canton. Further molecular investigations on the distribution of G6PD genotypes in the Taiwan aborigines, particularly on whether the G6PD Vanua Lava mutation exists or not, and on how deep this variant is spreading into Filipino populations, are awaited for understanding gene flows during Indonesian expansions.

## Conclusions

Based on published reports and our molecular epidemiological studies of Southeast Asian countries, specific features of distribution of G6PD deficiency genotypes in each community and country are discussed. Particularly, in Indonesia, the frequency distribution of genotypes was distinct between western and eastern Indonesian populations, suggesting two different gene flows during expansions of Indonesian peoples.

## Data Availability

Not applicable.

## Abbreviation

G6PD: Glucose 6 phosphate dehydrogenase
